# Formant-Based Recognition of Words and Other Naturalistic Sounds in Rhesus Monkeys

**DOI:** 10.3389/fnins.2021.728686

**Published:** 2021-10-29

**Authors:** Jonathan Melchor, José Vergara, Tonatiuh Figueroa, Isaac Morán, Luis Lemus

**Affiliations:** ^1^Department of Cognitive Neuroscience, Institute of Cell Physiology, Universidad Nacional Autónoma de México, Mexico City, Mexico; ^2^Department of Neuroscience, Baylor College of Medicine, Houston, TX, United States

**Keywords:** Psychophysics, long-term memory, formants, auditory discrimination, non-human primate (NHP)

## Abstract

In social animals, identifying sounds is critical for communication. In humans, the acoustic parameters involved in speech recognition, such as the formant frequencies derived from the resonance of the supralaryngeal vocal tract, have been well documented. However, how formants contribute to recognizing learned sounds in non-human primates remains unclear. To determine this, we trained two rhesus monkeys to discriminate target and non-target sounds presented in sequences of 1–3 sounds. After training, we performed three experiments: (1) We tested the monkeys’ accuracy and reaction times during the discrimination of various acoustic categories; (2) their ability to discriminate morphing sounds; and (3) their ability to identify sounds consisting of formant 1 (F1), formant 2 (F2), or F1 and F2 (F1F2) pass filters. Our results indicate that macaques can learn diverse sounds and discriminate from morphs and formants F1 and F2, suggesting that information from few acoustic parameters suffice for recognizing complex sounds. We anticipate that future neurophysiological experiments in this paradigm may help elucidate how formants contribute to the recognition of sounds.

## Introduction

Non-human primates (NHP) identify conspecific vocalizations (Rendall et al., 1996; Jovanovic et al., 2000; Ceugniet and Izumi, 2004; Belin, 2006) that inform troop members about food quality (Hauser, 1998; Slocombe and Zuberbühler, 2006) or nearby predators (Seyfarth et al., 1980b). These communication abilities are likely to rely on the activity of vocal recognition brain areas, homologous in humans and macaques (Petkov et al., 2008; Leaver and Rauschecker, 2010; Ortiz-Rios et al., 2015; Belin et al., 2018). However, how different acoustic parameters contribute to the recognition of sounds in NHP is not fully understood.

The literature points to periodicity (i.e., the fundamental and harmonic frequencies at which the vocal folds vibrate during phonation) and temporal envelope as possible cues for vocal recognition (Stevens, 1983; Chandrasekaran et al., 2011; Mesgarani et al., 2014; Brewer and Barton, 2016). Also important to recognition are the prominences in the spectral envelope, formant frequencies, that vary with changes in the shape of the supralaryngeal tract (e.g., jaw height and tongue protrusion) and the length of the individuals’ vocal tract (Remez et al., 1981; Lieberman and Blumstein, 1988; Rendall et al., 2004; Ghazanfar and Rendall, 2008; Ackermann et al., 2014).

First formant (F1) and formant 2 (F2) have been shown to be important for the identification of vowels in human languages (Peterson and Barney, 1952; Remez et al., 1981; Lieberman and Blumstein, 1988; Hillenbrand et al., 1995). Behavioral studies on baboons (*Papio anubi*), vervet monkeys (*Chlorocebus pygerythrus*), and Japanese monkeys (*Macaca fuscata*) have shown that the monkeys can use formants to discriminate synthetic vowels (Hienz and Brady, 1988; Sinnott, 1989; Sinnott and Kreiter, 1991; Sommers et al., 1992; Hienz et al., 2004). In addition, evidence suggests that rhesus macaques (*Macaca mulatta*) spontaneously perceive changes in formants (Fitch and Fritz, 2006), possibly for recognizing individuals by body size, gender, or age (Sinnott, 1989; Fitch, 1997; Rendall et al., 1998; Bachorowski and Owren, 1999; Smith and Patterson, 2005; Ghazanfar et al., 2007; Furuyama et al., 2016).

However, it has not been tested whether formants contribute to the discrimination of complex sounds, including words in macaques. We trained two rhesus monkeys to discriminate sounds learned as target (T) or non-target (NT). After training, we challenged the monkeys to discriminate morphs of T and NT and F1, F2, or F1F2-pass filters. Our results show that macaques are not only capable of storing numerous sounds in their long-term memories but that they also discriminate sounds embedded in morphs or from formant-pass filters. We anticipate that future neural recordings in this paradigm may explain the neuronal mechanisms of acoustic recognition.

## Materials and Methods

### Animals and Experimental Setup

Two adult rhesus macaques (*M. mulatta*; one 13 kg, 10-year-old male, and one 6 kg, 10-year-old female) participated in this study. The animals inhabited an enriched facility that allowed interactions with other monkeys. The macaques were restricted to water only for 3 h before experimental sessions. However, afterward, they received water *ad libitum*. The monkeys performed ∼1,000 trials for 3 h a day (4–5 days per week). Experiments took place in a soundproof booth where a macaque remained sitting on a primate chair, 60 cm away from a 21” LCD color monitor (1,920 × 1,080 resolution, 60 Hz refresh rate). A Yamaha MSP5 speaker (50 Hz–40 kHz frequency range) was set 15 cm above and behind the monitor to deliver sounds at ∼60 dB SPL measured at the monkeys’ ear level. Additionally, a Logitech^®^ Z120 speaker was situated directly below the Yamaha speaker to render white background noise at ∼50 dB SPL. Finally, a metal spring lever positioned at the monkeys’ waist level captured their responses.

### Behavioral Task

**FIGURE 1:**
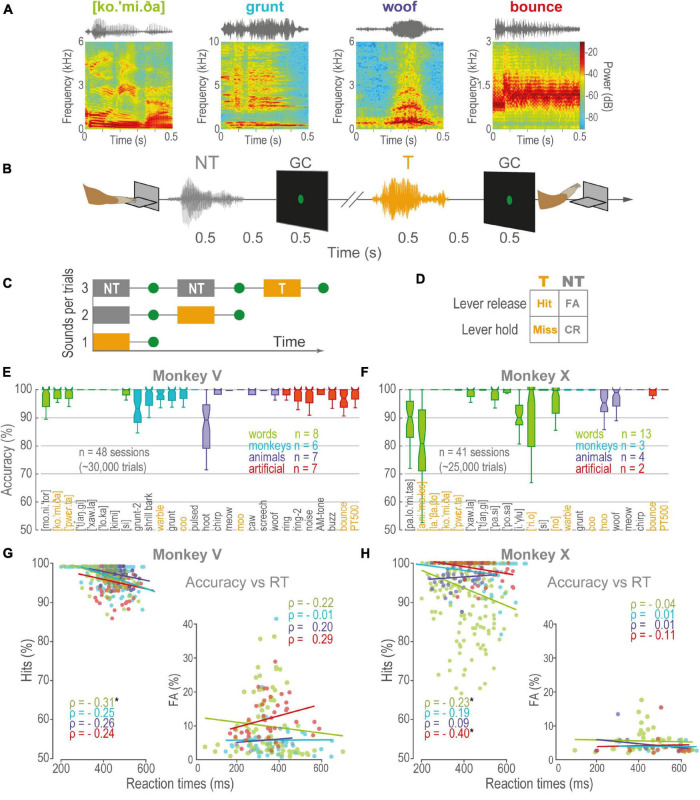
Acoustic discrimination task and performance. **(A)** Spectrograms of four acoustic categories: Green, words, Cyan, conspecific monkey vocalizations, Purple, vocalizations of animals, Red, artificial sounds. **(B)** The sequence of events in a trial: First, the monkey pressed a lever to start. After a variable period (0.5–1 s), a playback of 1–3 sounds commenced. Each sound was followed by a 0.5 s delay and a 0.5 s go-cue (GC). The monkey obtained a liquid reward for releasing the lever within 0.8 s of GC of sounds learned as T, but not during NT sounds. **(C)** Trials consisted of 0–2 NT followed by a T. **(D)** Outcomes of behavior. **(E,F)** Boxplots of the performance of monkeys V and X, respectively, during the discrimination of learned sounds. Orange, T, Gray, NT, other colors follow the color code for categories in **(A)**. Boxplot edges correspond to the 25th and 75th percentiles, central lines, medians. The vertical lines cover ± 2.7 SD. **(G)** ρ, Spearman’s Rho correlations between RT as a function of accuracy for Monkey V, during Hits (left panel), and FA (right panel). Linear regressions are visual comparisons of the correlations. Each dot is a session, same color code as in **(E,F)**. **(H)** Same as in **(G)**, but for monkey X. Asterisks are categories whose rho correlations were significant, *p* < 0.005.

We trained two rhesus monkeys (V and X) to discriminate learned sounds from various categories (Figure 1A). Each trial began with a gray circle at the center of the screen, indicating the monkey to press and hold down the lever in order to start a sequence of 1–3 sounds. Each sound lasted 0.5 s and was followed by a 0.5 s delay and the delay by a 0.5 s green go-cue (GC; Figure 1B). The probability of a T in a trial was: p (T| position_1) = 1/3, p (T| position_2) = 1/2, and p (T| position_3) = 1 (Figure 1C). Thus, trials of 1–3 sounds were presented pseudorandomly and with the same probability. The four possible outcomes of the behavior are illustrated in Figure 1D. To obtain a juice reward, the animal was required to keep down the lever throughout 0–2 NT (i.e., correct rejections, CR) and release within 0.8 s of the onset of the T GC (Hit). Releases before this period counted as false alarms (FA), causing the trial to be aborted. On the other hand, to release after the T GC window computed as a Miss. The task was programmed in LabVIEW 2014 (SP1 64-bits, National Instruments^®^).

### Acoustic Stimuli

**FIGURE 2:**
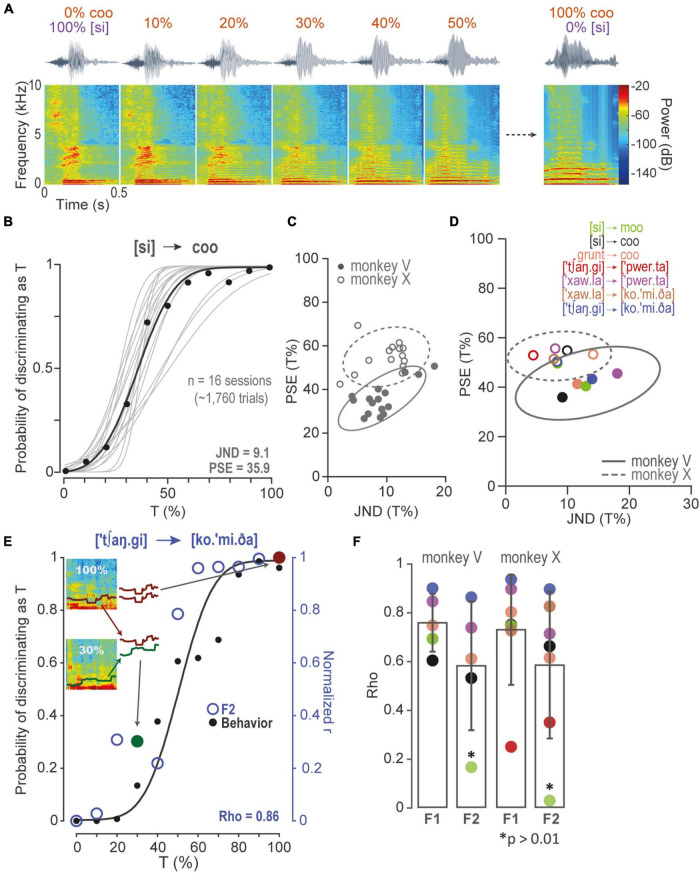
Discrimination of morphs and correlations between performance and formants. **(A)** Some spectrograms of the [si] to coo morph-line continua. The NT [si], i.e., the Spanish word for “yes,” morphed gradually in steps of 10 to 100% T “coo” monkey call. **(B)** PF of the probability of recognizing a morph as T. Gray lines, PFs of sessions of monkey V performing in set in **(A)**. Black dots, mean performance of all sessions in each morph. Black line, overall PF performance. **(C)** 2D-Gaussian fits of JND as a function of PSE, for PFs in **(B)**, and for monkey X. **(D)** 2D-Gaussian fits of centroids in **(C)** and the other morphing sets. **(E)** Spearman’s Rho correlation of monkey V performance (black dots) in a morphing set, and the distribution of Pearson’s *r* correlations (blue open circles). In this example, each open circle resulted from correlating F2 modulated in 100% T vs. F2 modulated in the other morphs. The red closed circle corresponds to the Pearson’s *r* of F2 in 100% T vs., again, F2 in 100% T (i.e., *r* = 1). Similarly, the green closed circle corresponds to the Pearson’s *r* of F2 in 100% T and F2 in 30% T. Notice that Pearson correlations are normalized in order to compare performance and F2 correlation probabilities directly. **(F)** Spearman’s Rho coefficients were obtained as in **(E)** for all morphing sets, monkeys, and F1 and F2. Same color code as **(D)**. Asterisks are non-significant rho correlations, *p* > 0.01.

The sounds were recorded in our laboratory or downloaded from free online libraries. They consisted of Spanish words (T = 6, NT = 10), monkey calls (T = 2, NT = 4), other animal’s vocalizations (T = 1, NT = 6), and artificial sounds (T = 2, NT = 5; Table 1). We normalized sounds to last 0.5 s, and we then resampled them to 44.1 kHz (cutoff frequencies, 100 Hz to 20 kHz) and finally equalized them (RMS; Adobe Audition^®^ version 6.0). The phonetic nomenclature for Spanish words was obtained using the automatic phonetic transcriptionist by Xavier López Morrás^1^. We also created the seven stimulus-morph-line continua (Figure 2A). In each morph-line, nine stimuli were spaced between an NT and a T. The morphs were created using the signal-processing software STRAIGHT (Speech Transformation and Representation based on Adaptive Interpolation of weighted spectrograms; Kawahara et al., 1999; http://www.wakayama-u.ac.jp/k̃awahara/STRAIGHTadv/index_e), following the protocol described by Chakladar et al. (2008) for mixing two sounds by relating salient spectral modulations. The monkeys obtained a reward for releasing the lever at morphs >50% T. However, the reward was delivered pseudorandomly for half the trials at 50% T in order to prevent the learning of that sound, which provided no real decisional criteria.

**TABLE 1:** Description of sounds.

	**Acoustic category**	**Sound ID**	**Description**
Target	Monkey	**coo**	Conspecific vocalization
		warble	Conspecific vocalization
	Words	**[ko.′mi.ða]**	Spanish word for food
		**[′pwεɾ.ta]**	Spanish word for door
		[a.ni.′ma.les]	Spanish word for animals
		[′ři.o]	Spanish word for river
		[no]	Spanish word for not
		[la.′βa.βo]	Spanish word for sink
	Animal	**moo**	Vocal sound of a cow
	Artificial	**bounce**	Bouncing tone
		PT500	Pure tone (500 Hz)
Non-target	Monkey	**grunt**	Conspecific vocalization
		grunt2	Conspecific vocalization
		shrill bark	Conspecific vocalization
		Pulsed	Conspecific vocalization
	Words	[′lo.ka]	Spanish word for crazy
		[kimi]	Spanish pseudoword
		**[′t∫aŋ.gi]**	Spanish pseudoword
		**[si]**	Spanish word for yes
		**[′xaw.la]**	Spanish word for cage
		[mo.ni.′tor]	Spanish word for monitor
		[′po.sa]	Spanish pseudoword
		[′pa.si]	Spanish pseudoword
		[i.′γlu]	Spanish word for igloo
		[pa.lo.′mi.tas]	Spanish word for popcorn
	Animal	meow	Cat vocalization
		chirp	Bird vocalization
		screech	Parrot vocalization
		caw	Crow vocalization
		**woof**	Dog vocalization
		hoot	Owl vocalization
	Artificial	AM-tone	1 kHz
		buzz	Mosquito whine
		ring	Cellphone ring tone
		ring2	Ring bell
		noise	Passband noise (1–4 kHz)

*Sounds in bold were selected for generating morphs and formant-pass sounds.*

Finally, we used a voice analysis app for Matlab (VoiceSauce version 1.36, http://www.phonetics.ucla.edu/voicesauce/; Shue et al., 2009) to generate formant-pass sounds (i.e., F1, F2, or F1F2). First, we derived F1 and F2 bandwidths in 25 ms windows every 1 ms. Then, we interpolated the bandwidths using Gaussian time-frequency representations (Elliott and Theunissen, 2009) and used an iterative inversion algorithm to synthesize the sounds^2^.

### Monkeys Training

We attempted diverse strategies to instruct the monkeys. Some details about instructions have been published elsewhere (Morán et al., 2021). However, some key elements were the following: First, the animals learned to press the lever in response to a gray circle and release it after a monkey coo vocalization, a 0.5 s delay, and a 0.5 s GC. Then, we introduced an NT, a delay, and a GC, and the monkeys had to wait and be still until T appearance. After learning a few T and NT, we introduced 0–2 NT to be presented before T. Once the monkeys learned the task sequence, they took only a few days to learn each new sound. The monkeys were not trained in the discrimination of morphs nor formant pass sounds; they were only exposed to those sounds at sessions reported here.

### Experimental Sessions

Each daily session consisted of one or two different experiments (e.g., the discrimination of learned sounds, morphs, or formants-pass filters). The morphs experiment consisted of one morph-line-continua set (e.g., [si]-moo or moo-coo). Each sound was presented randomly across trials and positions until repeated at least 10 times. The morphs were presented in the first position, where the probability of encountering a T was the lowest. However, the formant-pass sounds were presented in the first and second positions to achieve enough repetitions per sound. Each set was presented in a block so that trials of different experiments were not intermingled.

### Analysis

After exposing the animals to diverse sounds, we arbitrarily selected 5 T and 5 NT to perform most experiments (Table 1, bold fonts). We used non-parametric tests (Kruskal–Wallis, Mann–Whitney, and Wilcoxon) to evaluate performance and reaction times (RT) as a function of categories, positions, and subjects. We created psychometric functions (PF) by fitting Gaussian cumulative distribution functions to performance at morphing sets in order to quantify perceptual biases.


$$P\,\left(\text{release}\right)=\frac{1}{\sigma \,\sqrt{2\,\pi }}\,\int_{-\infty }^{x}\text{exp}\,\left(-\frac{{\left({\text{T}}_{\%}-\mu \right)}^{2}}{2\,\sigma^{2}}\right)$$

Where T% corresponds to T proportion in a morph, “μ,” is the point of subjective equality (PSE, or the morphing value at 50% chance of perceiving a T), and “σ” (STD) or just noticeable difference (JND, or the proportion to differentiate NT from T 84% of the times; σ = 1; Duarte and Lemus, 2017; Duarte et al., 2018). For all PF, *Q* > 0.05, *Q* = Γ(0.5●χ2, 0.5●v); where Γ = upper incomplete gamma function, χ2 = chi-square, and v = degrees of freedom (Press et al., 2007).

To evaluate performance throughout sessions of morphs, we fitted a 2D-gaussian of all PSE vs. their corresponding JND. Figure 2C compares both monkeys performing in all [si]-coo sessions. Figure 2D shows 2D-Gaussians to the centroids of all the other sets (Supplementary Figure 2B).

To quantify the contribution of each formant to the discrimination of morph-line stimuli, we calculated the similarity of each formant (F1 and F2) at each morph step to the same formant for the 100%-T stimulus. Similarity was quantified as Pearson’s *r*. These values were then correlated, Spearman’s rho, with the observed probability of identifying each stimulus in the morph line as a T (see Figures 2E,F).

We analyzed data using customized algorithms in MATLAB^®^ version 8.5.0.1, R2015a, The Mathworks, Inc.

## Results

The monkeys performed in a task consisting of discriminating as T or NT numerous sounds (*n* = 36, T = 11, NT = 25; Figures 1E,F). After instruction, we did three independent experiments: (1) the discrimination of learned sounds, (2) morphs, and (3) formant-pass filters.

### Rhesus Monkeys Learn and Discriminate Complex Sounds

The monkeys V and X discriminated the learned sounds above 50 % chance (V: *n* = 28; X: *n* = 22; Hits median: V = 0.97, X = 0.96; CR median: V = 0.98, X = 0.96; one-sample Wilcoxon signed-rank test, median = 0.75, Z [V_Hits] = 10.41, Z [V_CR] = 8.51, Z [X_Hits] = 9.63, Z [X_CR] = 7.87; *p* < 0.001). The animals did not show significant biases for any sound or category (Supplementary Figures 1A,B; pairwise Wilcoxon rank-sum test, false discovery rate corrected for multiple comparisons using the Benjamini-Hochberg procedure; *q*-value = 0.01). Despite the differences between the monkeys (V, X), the categories (T, NT), and the stimulus position (1st, 2nd, 3rd), mean performance was consistently above 90% accuracy (Supplementary Figures 1C,D). In general, monkey X was faster than V. However, there were only significant correlations between accuracy and RT for monkey X, with discriminating synthetic sounds and both monkeys discriminating words (Figures 1G,H and Supplementary Figures 1E,F). Overall, these results indicate the monkeys could learn and discriminate sounds of different categories.

### The Discriminations of Morphs Correlated With First Formant and Second Formant Modulations

To measure the monkeys’ capacity to discriminate sounds, we tested them in seven sets consisting of morphs of T and NT in different proportions. Figure 2A illustrates the NT [si] (i.e., the Spanish word for “yes”) gradually morphing to a T monkey “coo” call. Figure 2B shows PFs of all sessions (*n* = 16) in which monkey V performed at [si] to coo set (see also Supplementary Figure 2A). To compare their behaviors, we fitted a 2D-gaussian to all JND vs. PSE derived from each PF (Figure 2C and Supplementary Figure 2B). Similarly, we fitted 2D-Gaussians to the centroids obtained from the 2D-gaussian distributions of all sets (Figure 2D). The mean of centroids of monkey V was 19.7 ± 8.7, 41.5 ± 7.5 (JND ± SD, PSE ± SD), and of monkey X, 12.9 ± 6.3, 52.7 ± 4.9 (JND ± SD, PSE ± SD). Monkey V showed some bias to discriminate morphs as T (pairwise Wilcoxon rank-sum test, Benjamini-Hochberg FDR correction, *q*-value = 0.01, Supplementary Figures 2C,D). Nevertheless, both monkeys discriminated morphs proficiently.

To further study the contribution of formants to the monkeys’ discriminations, we calculated Spearman’s rho correlations between performance and F1 and F2 modulations to test the hypothesis that the probability of discriminating a morph as T was proportional to the correlation between the formants of the morphs and of 100% T. Figure 2E presents a PF and the distribution of the normalized Pearson’s *r* correlations along the morph-line continua. In this example, F2 correlated significantly to the probability of recognizing sounds as T (Spearman’s Rho, *p* < 0.01; see Supplementary Figure 2E for all morphing set). Figure 2F shows that F1 correlated with both of the monkeys’ performance in all morphing sets, whereas F2 correlated in 4 out of 5 sets for monkey V and 6 out of 7 for monkey X (Spearman’s Rho, *p* < 0.01).

### The Monkeys Discriminated Sounds Comprised of First Formant and Second Formant-Pass Filters

**FIGURE 3:**
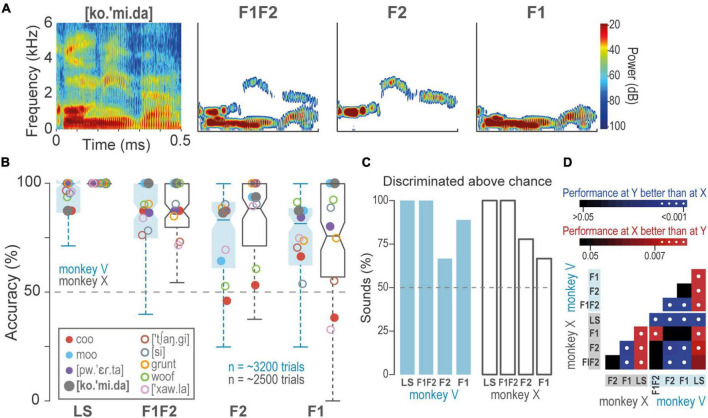
Discrimination of formant-pass sounds. **(A)** Spectrograms of T [ko.’mi.da] and F1F2, F2, and F1-pass sounds. **(B)** Boxplots of accuracy during the discrimination of formant-pass filters compared to the discrimination of the learned sounds (LS). Dash line, performance at chance = 50%. Closed circles, T, open circles NT. **(C)** Percentage of learned and formant-pass sounds discriminated above chance (Wilcoxon signed-rank test, *q* = 0.01). Dash line, performance at the chance. **(D)** *p*-values of multiple pairwise comparisons of sounds and monkeys. Color gradients indicate *p*-values, white dots, significant differences.

We presented the monkeys with F1, F2, and F1F2-pass filters synthesized from the learned sounds (Figure 3A). Figures 3B,C shows that both animals discriminated above chance most of the sounds, i.e., F1, 70.1% ± 14 (mean ± SD), F2, 72.6 ± 21, and F1F2, 79.2 ± 12.2. However, performance was significantly lower than during the discrimination of the learned sounds: Learned > F1F2 > F2 > F1 (Benjamini-Hochberg and FDR correction for multiple Wilcoxon signed-rank test comparisons; *q*-value = 0.01; Figure 3D). These results suggest that formants F1 and F2 provide relevant information about sounds.

## Discussion

We have presented evidence of the capacity of rhesus monkeys to learn and discriminate sounds from a broad range of frequencies and temporal modulations and corroborated that they are capable of discriminating morphs between pairs of sounds (Tsunada et al., 2011).

### Rhesus Macaques Have Long-Term Memories of Complex Sounds

Evidence of long-term memory of ethological sounds in monkeys is restricted to conspecific vocalizations (Seyfarth et al., 1980a). In the present study, we demonstrate that rhesus macaques can discriminate non-conspecific vocalizations and other naturalistic sounds. This perceptual ability may depend on circuits of acoustic categories, whose projections to motor areas could serve as feedback for vocal learning in species such as NHP and birds (Takahashi et al., 2017; Moore and Woolley, 2019; Zhao et al., 2019). It has been proposed that the learning of sounds in NHP is genetically determined (Brockelman and Schilling, 1984; Owren et al., 1992; Zador, 2019). In such a scenario, genetically programmed circuits should admit inclusions of non-ethological sounds as those that our monkeys learned.

In our task, learning consisted of associating two behaviors with diverse sounds, including conspecific vocalizations that may have had stereotyped responses. Similar associations to sounds have been reported previously for other communicating animals (Town et al., 2018; Saunders and Wehr, 2019; Yu et al., 2020). An important open question here is whether storing new sounds in long-term memory is achieved by nesting them to homophones (Chomsky, 1959). Consistent with previous reports, the training of our monkeys was more tenuous and prolonged than in visual or tactile tasks (Colombo and D’Amato, 1986; Colombo and Graziano, 1994; Wright, 1999, 2007; Fritz et al., 2005; Lemus et al., 2009a; Scott et al., 2012; Rajalingham et al., 2015). Therefore, acoustic learning based on nesting is unlikely since it would be possible to incorporate new sounds into existing circuits quickly. Alternatively, learning may depend on context (e.g., sentences), which, compared to humans, may be limited in macaques.

Did the monkeys learn whole sounds or only some segments? A possibility is that the animals learned only a chunk of sounds rather than all spectrotemporal modulations. Functional magnetic resonance imaging and electrocorticography studies in humans suggest that the representations of sounds start by phonetic relationships at the lateral bank of the auditory cortex (Chang et al., 2010; Obleser et al., 2010; Mesgarani et al., 2014). In macaques, neurons of the lateral belt respond to “monosyllabic” conspecific vocalizations of various broadband frequencies (Rauschecker et al., 1995) processed hierarchically along the superior temporal gyrus (Leaver and Rauschecker, 2010; Ortiz-Rios et al., 2015; Belin et al., 2018) up to the prefrontal cortex (Romanski et al., 1999; Rauschecker and Romanski, 2011). In our task, the animals were exposed to multisyllabic words, which were arguably learned in only the first or last portions. This possibility would concur with the idea of macaques being only capable of processing single units of sound, such as their vocalizations. Previous reports suggest that macaques use all available information to discriminate acoustic flutter (Lemus et al., 2009a, b). Those sounds consisted of periodic trains of pulses that might not have required the monkeys to listen entirely in order to discriminate. In our paradigm, sounds also lasted 0.5 s; however, sounds consisted of dynamical spectral modulations that the monkeys likely attended to in order to accumulate evidence and to improve performance (Brunton et al., 2013).

Ng et al. (2009) exposed macaques to complex sounds similar to ours in a match-to-sample task. In contrast to our results, they found that the animals performed better for conspecific calls than for other categories. This inconsistency may derive from differences between the short-term memory they tested and the long-term memory explored in our task. Similarly, in a delayed match-to-sample task (Scott et al., 2012), performance depended on presenting 0–2 distractors in a trial (i.e., 91, 73, and 39%, respectively). The authors concluded that this detriment was due to the number of distractors interfering with working memory. Again, performance was not affected in our study despite the position of sounds in a trial or ethological relevance. Future studies may determine differences in mechanisms and anatomical representations of short- and long-term memory in NHP (Munoz-Lopez et al., 2010; Muñoz-López et al., 2015; Fritz et al., 2016).

### Rhesus Monkeys Discern Categories From Acoustic Mixtures

We exposed the monkeys to acoustic morphs of T and NT to explore their discrimination thresholds. Our results are consistent with previous reports in humans categorizing monkey calls (e.g., coos, grunts, and harmonic arches; Furuyama et al., 2017; Jiang et al., 2018) and the /a/ vowel (Chakladar et al., 2008), suggesting that macaques possess an acoustic perception similar to that of humans. Similarly, Tsunada et al. (2011) trained macaques to discriminate morphs of the syllables /bad/ and /dad/ to study the neuronal correlates of acoustic categorization. They found that the neurons of the auditory belt area presented categorical responses to the graded mixtures, meaning that those neurons correlated with decisions rather than the perception of acoustic parameters. Therefore, to explore the impact on acoustic perception of parameters such as F1 and F2 formants, related to the recognition of vowels in humans (Peterson and Barney, 1952; Remez et al., 1981; Lieberman and Blumstein, 1988; Hillenbrand et al., 1995), we computed correlations between the psychometric curves in monkeys and those features. Our results show that F1 and F2 indeed correlated with behavior. Something noteworthy to mention is that regardless of the fact that the animals learned only some sounds, they nevertheless could discriminate morphs to which they were exposed on only a few occasions. In other words, the monkeys discriminated from modified information of learned sounds, suggesting that perception is invariant. In any case, this result cannot rule out that other acoustic features contribute to perception (Stevens, 1983; Brewer and Barton, 2016).

### Monkeys Discriminate Complex Sounds Based on Formant Frequencies

To test whether formants sufficed for discriminations, we presented the monkeys with formant-pass sounds. We found that formants indeed sufficed. Furthermore, F1 and F2 combined improved performance as compared to F1 and F2 alone. However, to further understand how formants participate in acoustic perception, an exciting control would be to present only the complementary information to F1- and F2-pass filters.

Since formants constitute the most energetic modulations in sounds, they may significantly shape neuronal circuits representing sounds. Here the hypothesis is that salient signals excite neurons in higher probability than other signals (at least in primary sensory areas). For instance, formants simultaneously activate neurons at different frequency bands of the auditory cortex. Those cells, in turn, could activate upstream neurons, creating circuits of acoustic representations (Hebb, 1949). Our findings suggest that formants contribute to the discrimination of complex sounds in macaques, perhaps like for humans in the perception of communication sounds (Remez et al., 1981; Fitch and Fritz, 2006; Ghazanfar et al., 2007; Furuyama et al., 2016, 2017).

## Data Availability Statement

The raw data supporting the conclusions of this article will be made available by the authors, without undue reservation.

## Ethics Statement

The animal study was reviewed and approved by Mexican Official Standard Recommendations for the Care and Use of Laboratory Animals (NOM-062-ZOO-1999) and the Internal Committee for the Use and Care of Laboratory Animals of the Institute of Cell Physiology, UNAM (CICUAL; LLS80-16).

## Author Contributions

JM and IM performed experiments. JM, JV, and LL analyzed data and prepared the figures. JM, TF, JV, and IM revised the manuscript. TF programmed the task. LL wrote the manuscript. All authors contributed to the article and approved the submitted version.

## Conflict of Interest

The authors declare that the research was conducted in the absence of any commercial or financial relationships that could be construed as a potential conflict of interest.

## Publisher’s Note

All claims expressed in this article are solely those of the authors and do not necessarily represent those of their affiliated organizations, or those of the publisher, the editors and the reviewers. Any product that may be evaluated in this article, or claim that may be made by its manufacturer, is not guaranteed or endorsed by the publisher.

## Footnotes

1
http://aucel.com/pln/transbase.html

2
http://theunissen.berkeley.edu/software.html

## Abbreviations

NHP: non-human primates
T: Target
NT: non-target
F1: first formant
F2: second formant
CR: correct rejections
FA: false alarms
GC: go-cue
RT: reaction time
PF: psychometric function
PSE: point of subjective equality
JND: just noticeable difference.
