# Supplementary material for: Formant-Based Recognition of Words and Other Naturalistic Sounds in Rhesus Monkeys
Source: Front Neurosci. 2021 Oct 29;15:728686. doi: 10.3389/fnins.2021.728686 (PMC8586527; doi:10.3389/fnins.2021.728686)
Supplement: Supplementary file 1 [file Image_1.pdf]

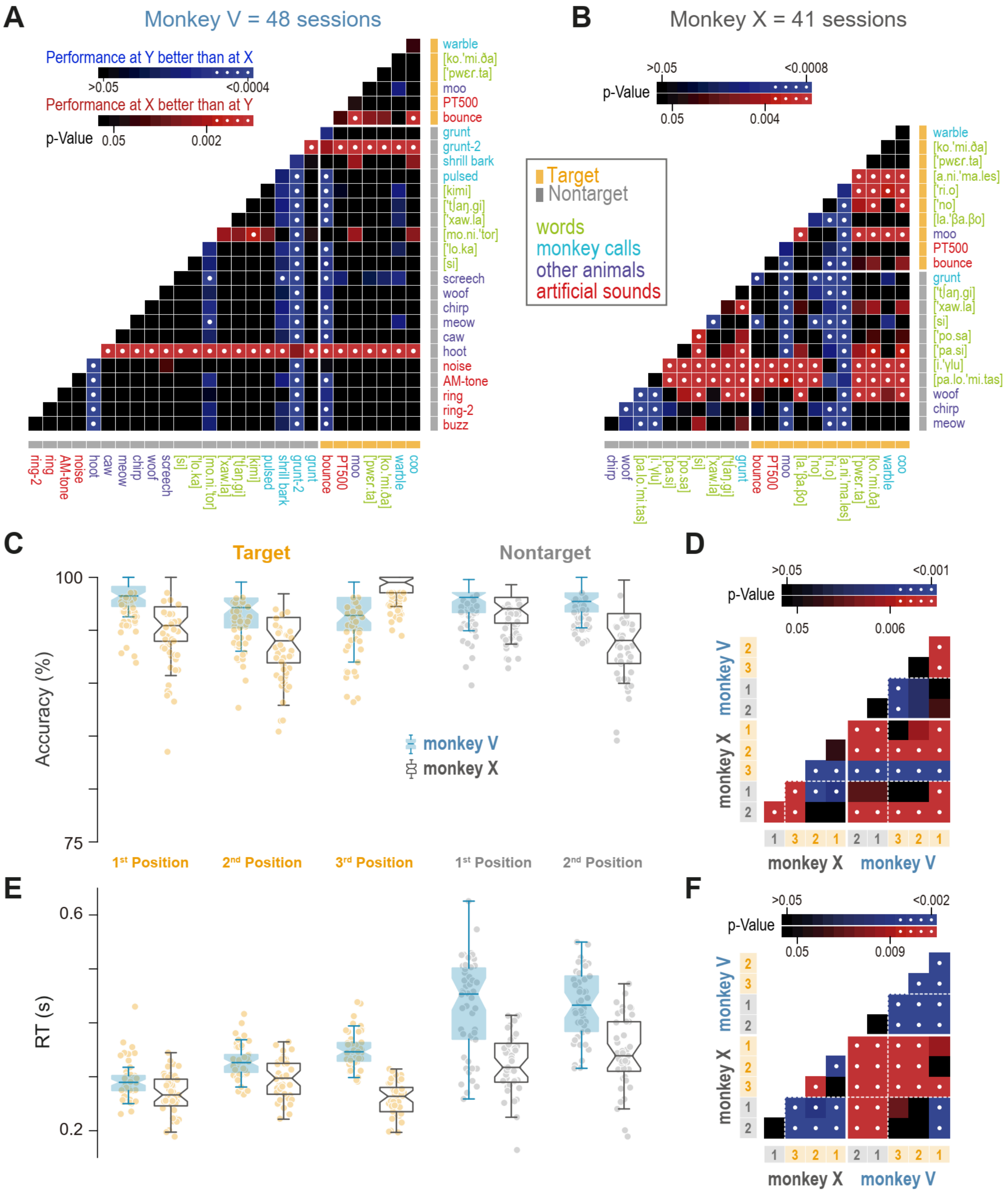

SUPPLEMENTARY FIGURE 1. Accuracy and RT comparisons across acoustic categories and monkeys. (A) Confusion matrix of p-values of multiple pairwise comparisons of monkey V accuracy during the discrimination of different acoustic categories. The intensity at the upper color bar is proportional to the p-value. White circles indicate significant differences. **(B)** Same as **A**, but for monkey X. **(C)** Monkeys' accuracy during the discrimination of T and NT presented in different positions. Each dot represents the mean performance in a session. Box plot edges correspond to the 25th and 75th percentiles, the central line to the median, and vertical lines ± 2.7 SD. **(D)** Confusion matrix of p-values of multiple comparisons of performance between monkeys, categories, and position. **(E)** Same as in **C** but for RT. **(F)** p-values of multiple pairwise comparisons of RT, same labels as panel **D**.

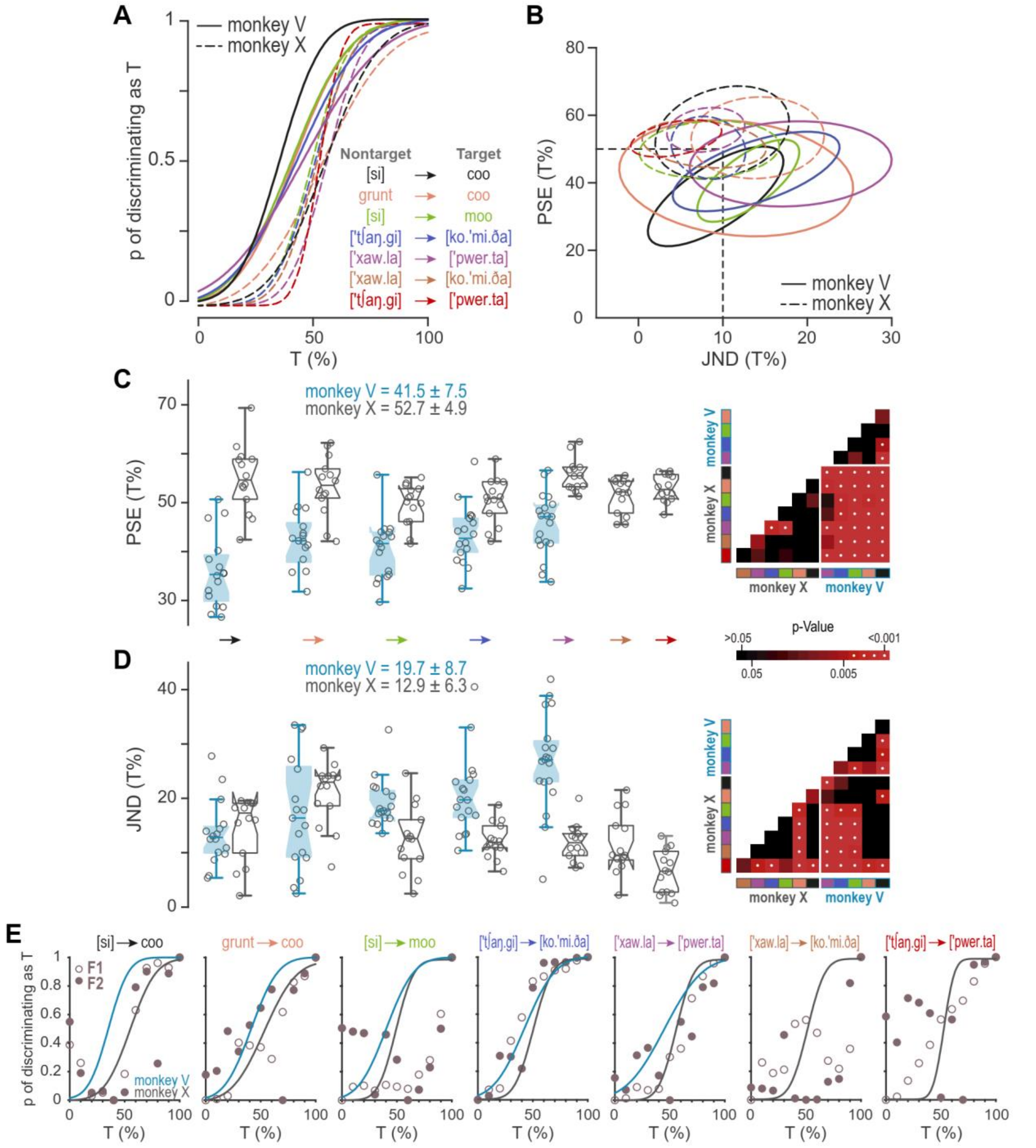

SUPPLEMENTARY FIGURE 2. Psychometric analysis of the discrimination of sounds. (A) PFs of both monkeys discriminating in different morphing sets. Each PF corresponds to the probability of discriminating as T the morphs at the abscissas. **(B)** 2D-Gaussian fits of PSE as a function of JND of both monkeys performing in all morphing sets. **(C)** Boxplots of both monkeys' PSE of all morphing sets. Box edges and lines follow the same convention as in Supplementary Figure **1C, E**. Same color code as **A.** Right panel, p-values of multiple pairwise comparisons of PSE. Color gradients indicate p-values, white dots, significant differences. **(D)** Same as in **C**, but for JND. **(E)** PFs and the distribution of the normalized Pearson's r correlations for formants along the morph-line continua.
